# High Speed AFM and NanoInfrared Spectroscopy Investigation of Aβ_1–42_ Peptide Variants and Their Interaction With POPC/SM/Chol/GM1 Model Membranes

**DOI:** 10.3389/fmolb.2020.571696

**Published:** 2020-09-09

**Authors:** Cecile Feuillie, Eleonore Lambert, Maxime Ewald, Mehdi Azouz, Sarah Henry, Sophie Marsaudon, Christophe Cullin, Sophie Lecomte, Michael Molinari

**Affiliations:** ^1^CBMN, CNRS UMR 5248, IPB, Université de Bordeaux, Pessac, France; ^2^LRN EA 4682, Université de Reims Champagne-Ardenne, Reims, France; ^3^Department of Chemistry, Université de Montréal, Montreal, QC, Canada

**Keywords:** high speed atomic force microscopy, nanoInfrared spectroscopy, peptide Aβ, model lipidic membranes, Alzheimer’s disease

## Abstract

Due to an aging population, neurodegenerative diseases such as Alzheimer’s disease (AD) have become a major health issue. In the case of AD, Aβ_1__–__42_ peptides have been identified as one of the markers of the disease with the formation of senile plaques via their aggregation, and could play a role in memory impairment and other tragic syndromes associated with the disease. Many studies have shown that not only the morphology and structure of Aβ_1__–__42_ peptide assembly are playing an important role in the formation of amyloid plaques, but also the interactions between Aβ_1__–__42_ and the cellular membrane are crucial regarding the aggregation processes and toxicity of the amyloid peptides. Despite the increasing amount of information on AD associated amyloids and their toxicity, the molecular mechanisms involved still remain unclear and require in-depth investigation at the local scale to clearly decipher the role of the sequence of the amyloid peptides, of their secondary structures, of their oligomeric states, and of their interactions with lipid membranes. In this original study, through the use of Atomic Force Microscopy (AFM) related-techniques, high-speed AFM and nanoInfrared AFM, we tried to unravel at the nanoscale the link between aggregation state, structure and interaction with membranes in the amyloid/membrane interaction. Using three mutants of Aβ peptides, L34T, oG37C, and WT Aβ_1__–__42_ peptides, with differences in morphology, structure and assembly process, as well as model lipidic membranes whose composition and structure allow interactions with the peptides, our AFM study coupling high spatial and temporal resolution and nanoscale structure information clearly evidences a local correlation between the secondary structure of the peptides, their fibrillization kinetics and their interactions with model membranes. Membrane disruption is associated to small transient oligomeric entities in the early stages of aggregation that strongly interact with the membrane, and present an antiparallel β-sheet secondary structure. The strong effect on membrane integrity that exists when these oligomeric Aβ_1__–__42_ peptides interact with membranes of a particular composition could be a lead for therapeutic studies.

## Introduction

Alzheimer’s disease (AD) is a neurodegenerative disease associated with progressive memory loss and dementia. Two types of abnormal amyloid aggregates are found in the patient’s brains, extracellular Amyloid β plaques containing Aβ peptides and intracellular neurofibrillatory tangles (NFT) composed of hyperphosphorylated Tau protein. Both are involved in a complex cascade of events leading to synapse dysfunction and neuronal death. The formation of senile plaques, one of the hallmarks of the pathology, results from the self-assembly of Aβ aggregates into fibers. As the major component of these plaques, the 42 amino-acid long amyloid peptide Aβ_1__–__42_ has been shown to be neurotoxic (Yasumoto et al., 2019) playing a role in the memory impairment and the other tragic syndromes associated with the disease. Aβ_1__–__42_ is considered as a biological key player in AD (Selkoe, 2001), but its fibrillization is not the only parameter involved in the AD disease. An overwhelming body of evidence in *in vitro* (Henry et al., 2015; Yasumoto et al., 2019) and *in vivo* (Fruhmann et al., 2018; Yasumoto et al., 2019) studies has highlighted the importance of Aβ_1__–__42_/membrane interactions and the crucial role that lipid bilayers play regarding fibrillogenesis and toxicity (Williams and Serpell, 2011).

As stated before, the aggregation state of the amyloid species is a crucial parameter in the amyloid/membrane interaction. Intermediate species of Aβ peptides in the fiber formation process, such as monomers, oligomers, protofibrils and fibers, have distinct cytotoxicities. The oligomer species have been identified as the most toxic species (Henry et al., 2015; Kepp, 2017; Bode et al., 2019; Limbocker et al., 2019; Yasumoto et al., 2019), and are often associated with a secondary structure of antiparallel β-sheets (Bobo et al., 2017; Henry et al., 2018). However the heterogeneity of amyloid species populations in biophysical studies is an obstacle to the precise understanding of the involved processes (Doig et al., 2017; Henry et al., 2018). Indeed, the aggregation state is often not controlled, leading to the observation of an average effect of several aggregation species (Matsuzaki, 2014; Bode et al., 2019) and therefore to a lack of reproducibility (Henry et al., 2015). The separate characterization of monomers, oligomers and fibers is required in order to fully understand the mechanisms of amyloid toxicity. In order to study in details the action mechanisms of toxic oligomers, an approach has been to separate fibrillization products by size-exclusion chromatography (Vignaud et al., 2013; Bode et al., 2019; Yasumoto et al., 2019). Gaining from such isolation of fibrillization species, the understanding of oligomer-induced cytotoxicity has recently benefitted from several advances. High molecular weight oligomers produce more plasma membrane damage than low molecular weight ones, with additional oxidative stress and membrane fluidity decrease (Yasumoto et al., 2019). It is, however, still unclear how the observed deleterious effects relate to the sequence of the amyloid peptide, its secondary structure, or its aggregation state.

Apart from the properties of the Aβ peptide, several studies also showed that the interactions of Aβ with lipid membranes are strongly modulated by several membrane parameters including its lipid composition and its organization and phase separation. Ganglioside GM1 is a preferential partner in the Aβ/membrane interaction, and Aβ species have been shown to accumulate on GM1 clusters, which alter the aggregation pathways of Aβ into the formation of toxic species (Matsuzaki, 2014; Dai et al., 2020). Interestingly, the formation of GM1 clusters is cholesterol-dependent, as a depletion in cholesterol in neuronal cells rescued them from Aβ accumulation and toxicity (Matsuzaki, 2014). Indeed, lipid composition influences the membrane nanoscale organization, as demonstrated *in vitro* on membrane models (Drolle et al., 2017). The presence of membrane domains was notably shown to modulate the amyloid/membrane interaction, with Aβ peptides interacting preferentially with liquid domains (Azouz et al., 2019). Membrane heterogeneity can also be induced by Aβ aggregation and binding, with a decrease of the lateral fluidity of the membrane (Sasahara et al., 2013). Different models of interaction have been proposed for Aβ-membrane interaction (Berthelot et al., 2013), from adsorption on the membrane to dissolution or insertion, formation of pores, ion channels, or raft-like structures (Drolle et al., 2014), while an uptake of lipids in Aβ aggregates (Sasahara et al., 2013) suggests lipid recruitment.

Though these studies bring valuable information to the field, questions still arise especially regarding the molecular mechanisms involved in AD, which remain elusive and require in-depth investigation at the local scale (Doig et al., 2017). The understanding of the structure of the amyloid species and of their interactions with membranes at the molecular scale could provide new insights leading to the development of novel therapies, especially considering that clinical trials focused on the removal or disassembly of amyloid plaques have not been successful so far (Panza et al., 2019). One limitation of the experiments classically performed in most studies (e.g., ThT fluorescence assays, Infrared absorption spectroscopy, Circular dichroism) is their lack of resolution leading to the characterization of averaged properties of amyloid peptides and of lipid membranes. Most of the time, different fibrillization forms of peptides (monomers, oligomers, fibrils) coexist in solution, with different structures, while a strong heterogeneity is observed in the membrane composition and organization, thus complicating a precise identification of the predominant mechanisms involved in AD. Among the different tools that could help decipher these mechanisms, Atomic Force Microscopy (AFM) is of particular interest due to its high resolution and its ability to work in liquid conditions (contrary to cryo-electron microscopy for instance). In particular, AFM allowed adding some interesting information regarding the fibrillization process of the peptides (Mastrangelo et al., 2006; Hane et al., 2014). Nevertheless, AFM is still limited by the speed of imaging acquisition and by the lack of information about the chemical properties of studied objects. Lately, these two drawbacks have been addressed through the development of High Speed AFM (HS-AFM), allowing real time experiments, and of nanoInfrared AFM (NanoIR) (Dazzi et al., 2010, 2012) and Tip-Enhanced Raman Spectroscopy (TERS) AFM (Bonhommeau and Lecomte, 2018) providing chemical and structural information at the nanoscale without the labeling used in super-resolution optical microscopy, which can affect the structures of labeled species and their interactions. HS-AFM has been used to address the questions of the dynamics of aggregation for Aβ_1__–__42_ (Watanabe-Nakayama et al., 2016) but also to study the interaction between lipid membranes and Aβ peptides in real time (Ewald et al., 2019). Besides, NanoIR is increasingly used for chemical and structural characterization of biological objects, notably providing insights on the evolution of proteic secondary structure during amyloid aggregation of josephin fibers (Ruggeri et al., 2015), lysozyme (Islam et al., 2020), α-synuclein (Zhou and Kurouski, 2020), or Aβ_1__–__42_ (Henry et al., 2018).

**FIGURE 1:**
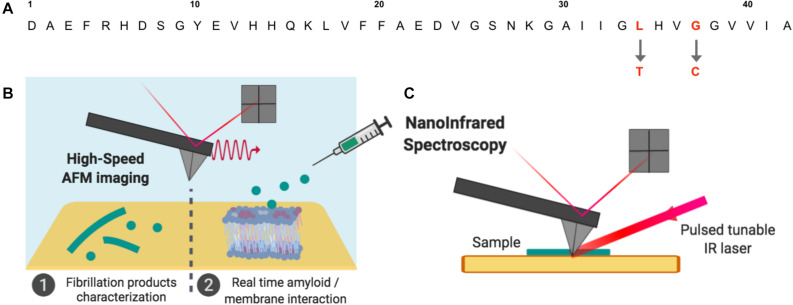
Aβ amyloid peptides and operating setups used in this study. **(A)** Three Aβ_1–42_ sequences were used: compared to Aβ_1–42_ WT, the Aβ_1–42_ L34T, and oG37C peptides present single mutations at position 34 and 37 respectively. **(B)** The fibrillation products obtained for the mutant peptides and their interactions with model lipid membranes were studied by High-Speed Atomic Force Microscopy (HS-AFM) in buffer medium. **(C)** Dried amyloid samples were also studied by NanoInfrared Spectroscopy, where the AFM cantilever detects thermal expansion of the sample after illumination by a tunable IR laser leading to local absorption IR spectra.

In this study, we are taking advantage of the latest developments in AFM using both NanoIR and HS-AFM to investigate at the nanoscale the link between aggregation state, structure and interaction with membranes in the amyloid/membrane interaction. The use of such experiments allows correlating in this original work morphology and structure of Aβ peptides of different toxicities and their interactions with model lipidic membranes of controlled composition. Based on previous work, three mutant Aβ peptides, L34T, oG37C and wild type (WT) Aβ_1__–__42_ peptides are used because of their differences in morphology, structure and assembly process. These well-controlled and characterized highly pure Aβ_1__–__42_ peptides variants present reproducible kinetic assemblies (Vignaud et al., 2013; Henry et al., 2015) ranging from very low to high toxicities in yeast compared to the WT peptide, with single mutations in the peptide’s sequence completely changing its fibrillization as well as its toxic behavior (Figure 1A). In comparison with the WT peptide, the substitution of an L residue in position 34 by a T (Aβ_1__–__42_ L34T peptide) leads to a decreased toxicity, whereas the replacement of a G residue in position 37 by a C (Aβ_1__–__42_ G37C peptide) leads to an increased toxicity. The purification of the later G37C peptide by size-exclusion chromatography leads to an oligomeric form, oG37C, coming from an “off-pathway” process (Vignaud et al., 2013; Henry et al., 2015). This oG37C oligomer is stable in solution as a 14-mer (Bobo et al., 2017), does not form fibers and shows the highest toxicity in yeast, which makes it the perfect model homogeneous oligomer to better understand the oligomer/membrane interaction with model membranes of controlled lipid compositions. Model membranes composed of Sphingomyelin (SM), 1-Palmitoyl-2-oleoylphosphatidylcholine (POPC), Cholesterol (Chol), and ganglioside GM1 have been used as they have previously been shown to have a strong interaction with the Aβ peptides (Ewald et al., 2019) leading to their disruption after addition of toxic peptides. In this paper, using NanoIR and HS-AFM, we try to answer the question whether the behavior of the mutant oligomer oG37C reflects the one of WT Aβ_1__–__42_ peptides, and whether it is possible to obtain a correlation between the toxicity of the peptides, their morphologies and structures, and their interactions with SM/PC/Chol/GM1 membranes. AFM and HS-AFM allow characterizing the mutant amyloid peptides and their fibrillization products at the nanoscale, as well as their interaction with model SM/PC/Chol/GM1 lipid membranes of controlled composition in real time (Figure 1B). NanoIR (Figure 1C) is used to locally probe the secondary structure of amyloid mutant peptides and their fibrillization products, as well as their accumulation on model membranes. Our observations show a correlation between secondary structure of the peptide, fibrillization kinetics and interaction with membranes.

## Materials and Methods

### Peptide Production

Aβ peptides were selected in *Saccharomyces cerevisiae*, produced in *Escherichia coli* and purified as previously described (D’Angelo et al., 2013). Following purification and dialysis, oG37C were isolated through size-exclusion chromatography (Vignaud et al., 2013). After quantification by Bradford protein assay, peptides were frozen in liquid nitrogen and stored at −80°C until further use.

### Fibrillization Study

Peptides were diluted in Tris(hydroxymethyl)aminomethane buffer (TRIS) (Tris 10 mM, NaCl 150 mM, DTT 5 mM, pH 7.4, referred to as TRIS buffer below) reaching a final peptide concentration of 20 μM, and were incubated at 37°C. Samples were collected regularly after homogenization by vortex in order to follow the morphological evolution of fibrillization species formed along the aggregation process on short and long time scales.

### Preparation of Supported Lipid Bilayers (SLB) and Multilayers

Lipids of interest were purchased from Avanti Polar lipids: 1-Palmitoyl-2-oleoylphosphatidylcholine (POPC), sphingomyelin (SM), cholesterol (Chol), and ganglioside GM1 (GM1). After dissolution in a solution of chloroform/methanol (4:1 v/v), the lipids were mixed in the following proportions: SM/POPC/GM1/Chol 20/20/40/20 (w%), homogeneized, evaporated under N_2(g)_ flux and kept in a dessicator overnight to remove any trace of organic solvent. The resulting lipid films were re-hydrated with TRIS buffer reaching a final concentration of 1.5 mg/ml of lipids. The lipid suspensions were vortexed until total dissolution and submitted to 5 freeze/thaw cycles. Large unilamellar vesicles (LUV) were then obtained by extrusion performed at 65°C using 100 nm diameter pores.

The LUV suspensions were diluted 5 times in 10 mM CaCl_2_, and 2 μL of the resulting solution were deposited onto freshly cleaved mica. Samples were incubated for 45 min at 60°C, rinsed and observed by HS-AFM in buffer. The samples were between 1 and 4 bilayers thick (∼ between 5 and 20 nm height).

### High-Speed AFM Imaging

A self-built High-Speed atomic force microscope (HS-AFM) similar to the one used by Ando et al. (2001) and provided by RIBM was used. Imaging was performed in tapping mode at room temperature, using Si_3_N_4_ rectangular cantilevers (BL-AC10DS-A2, Olympus) with a nominal spring constants of ∼0.2 N.m^–1^, oscillation frequency of ∼630 kHz in aqueous medium and an electron beam deposited amorphous carbon tip grown on the original tip. The free oscillation amplitude of the cantilever was adjusted to ∼2 nm with a setpoint amplitude set at approximately 85% of the free oscillation amplitude. The images and videos were analyzed using the Falcon Viewer analysis software, applying a first order polynomial filter to remove sample tilt when necessary. Topographical sections were extracted from the images.

For the morphological observation of fibrillization products, samples were prepared by depositing on freshly cleaved mica 2 μL of amyloid peptides collected at different times in the aggregation process. After 5 min of incubation, samples were rinsed with 6 μL of TRIS buffer, and subsequently placed on the AFM setup for observation in liquid. Because of the slow kinetics of fiber formation, the process was not followed continuously by HS-AFM but images were recorded at given times. Though the mica substrate has an impact on membrane properties, notably on lipid diffusion (Scomparin et al., 2009), it is a standard substrate used in AFM-based studies because of its planarity and has therefore been chosen in our study in order to reproduce previous results and use them as a reference point.

In order to study the impact of different Aβ mutant peptides on supported lipid bilayers, a SLB was first prepared and imaged by HS-AFM. After identification of a zone of interest, 6 μL of freshly thawed peptides at a concentration of 20 μM were injected in the HS-AFM fluid cell (final volume, 80 μL) while simultaneously adjusting the setpoint amplitude to counter any perturbations induced by the injection. Final concentration of peptide in the experimental volume was 1.5 μM.

### NanoInfrared Measurements

NanoInfrared AFM experiments were performed using a NanoIR2 system (Brüker, United States), with an OPO laser covering the range from 890 to 3600 cm^–1^ to acquire full IR spectra or a QCL laser covering the range from 1000 to 2000 cm^–1^ when focusing on the amide I bands. The measurements were carried out in contact mode (OPO laser) or in resonant contact mode (QCL laser) using a gold-plated silicon nitride AFM probe with an elastic constant of 0.07–0.4 N/m and nominal radius of 20 nm (model PR-EX-nIR2, Bruker, United States). The different images and spectra were analyzed using the Analysis Studio software (Brüker, United States). Once the fibrillization products were observed in liquid on mica (see previous HS-AFM experiments), the same samples were dried and analyzed in NanoIR to be sure to study the same objects. No changes in the morphology were observed in the fibers or the oligomers during the drying process. IR spectra were collected on isolated structures of the different peptides (L34T, WT, and oG37C) using the spectroscopic mode of the nanoIR. For each peptide, at least 50 spectra were recorded and analyzed. The same protocol was used to analyze the lipid SM/PC/Chol/GM1 bilayers before and after injection of the different peptides. After injection of the peptides as previously described for HS-AFM experiments and a 4 h incubation in humid conditions, the samples were imaged in order to check for the persistence of lipid layers. They were then rinsed with pure water so as to get rid of the non-interacting peptides present in the solution, dried in air and analyzed in NanoIR after finding a proper spot with visible layers on the sample surface. At least 50 spectra were then acquired on different spots for each sample.

## Results

### Morphology of Aβ_1__–__42_ Peptides and Their Fibrillization Products

**FIGURE 2:**
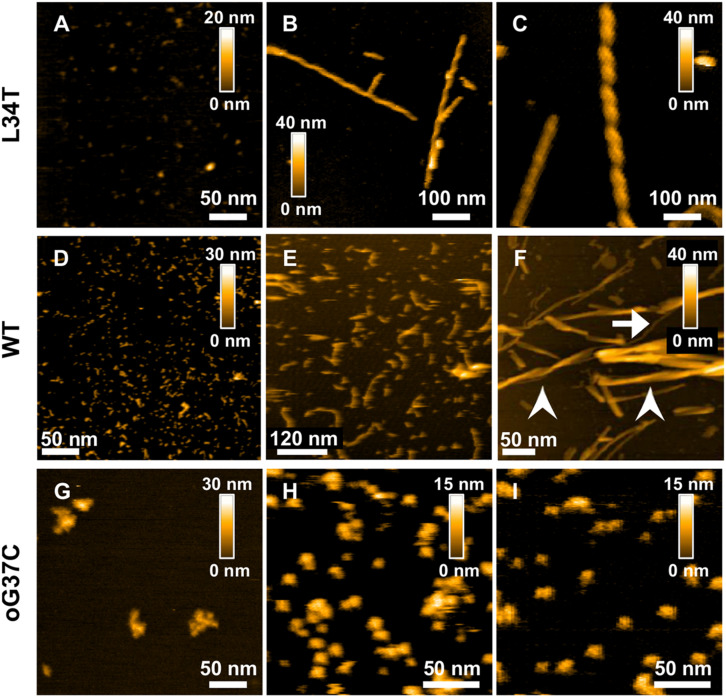
Morphology of Aβ_1–42_ WT and mutant peptides and their fibrillation products revealed by AFM height images obtained for L34T **(A–C)** WT **(D–F)**, and oG37C **(G–I)** peptides at different times in the fibrillation process, with images acquired at day 0 **(A,D,G)**, day 1 **(B,H)**, day 2 **(C,E,I)**, and day 7 **(F)**. All peptides are incubated at 20 μl. White arrows indicates unique fibers, arrow-heads indicates fiber bundles.

First, the aggregation state of each different amyloid peptide at the initial time of thawing was assessed, allowing to confirm the monomeric/oligomeric state of the peptide at the moment of our membrane interaction experiments. HS-AFM was used to observe freshly thawed peptides deposited on mica (Figures 2A,D,G). The Aβ_1__–__42_ WT peptides, as well as Aβ_1__–__42_ L34T present similar initial morphologies with small globular species below 10 nm in diameter on the mica substrates. In the case of the oligomer Aβ_1__–__42_ oG37C, larger globular aggregates are preferentially observed with sizes ranging from 20 to 50 nm in diameter, with occasional small globular objects (5–10 nm) still present, corresponding to previously observed isolated oligomers (Bonhommeau et al., 2017; Ewald et al., 2019).

**FIGURE 3:**
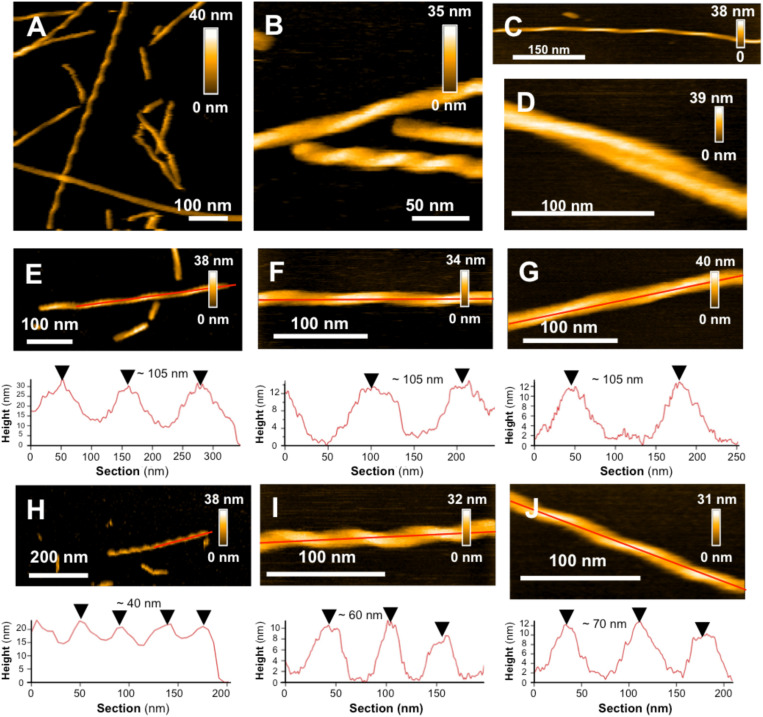
L34T polymorphism and its evolution in time SHOWN BY HS-AFM images of L34T samples collected and observed after 7 **(A,B)** and 10 days fibrillation **(C,D)** showing several morphologies, with straight twisted fibers with short and long cross-over distances, curved fibers and mature filaments. **(E–G)** Evolution in time of periodic fibers with long and **(H–J)** shorter cross-over distance, acquired at days 1, 7 and 10, respectively. Height images are represented with a topographic cross section taken along the red line.

Then the aggregation kinetics of the different mutant peptides was followed and compared to the WT peptide by imaging their fibrillization products at different incubation times, thus complementing previous work using ThT fluorescence and cryo-electron microscopy (Vignaud et al., 2013; Henry et al., 2015). Marked morphological differences were observed between fibrillization products of the different mutant Aβ_1__–__42_ peptides. Rapid fiber formation for the Aβ_1__–__42_ L34T peptide is evidenced (Figures 2A–C), with the formation of well-separated long straight fibrils (>600 nm) within 24 h (Figures 2B,C), with highly periodic morphologies, and regularly spaced cross-over distances. This twisted ribbon morphology is the most frequent in our observations, in agreement with literature on amyloid polymorphism (Adamcik and Mezzenga, 2018). At day 7 of incubation (Figures 3A,B,F,I), L34T presents an increasing number of fibers, with a majority of long well-separated straight fibers. In contrast, after 2 days the Aβ_1__–__42_ WT peptide forms only short fibrils (∼100 nm in length) with curved or random morphologies, and abundant globular aggregates as well (Figure 2E). At day 7 the fibers obtained for the WT (Figure 2F) are thicker and shorter than the L34T ones, and assemble majoritarily into bundles of twisted short fibers, in accordance with literature (Fitzpatrick et al., 2013; Nirmalraj et al., 2020), though occasional single fibers are observed (example pointed by the white arrow on Figure 2F). Contrary to both WT and L34T peptides, the Aβ_1__–__42_ oG37C oligomer does not aggregate further compared to our initial assessment (Figures 2H,I), and we still observe similar globular aggregates (20–50 nm in diameter), though more abundant on the mica substrate. This confirms that oG37C is stable as an oligomeric form and no fibril or mature fibers are formed.

### L34T Amyloid Polymorphism

In a single amyloid sample, it is common to observe different types of morphologies, which can either be in equilibrium or come from distinct intermediates on the aggregation pathway (Cendrowska et al., 2020). Polymorphism is of high interest as distinct morphologies have been shown to lead to different patterns of toxicity or spreading (Cendrowska et al., 2020). Our observations are based on homogeneous starting stocks of peptides with similar growth conditions, allowing to show morphological heterogeneity between mature fibers of Aβ_1__–__42_ WT and L34T, implying an influence of the peptide’s sequence in the morphology. High-resolution AFM imaging also allowed us to go further in the morphological characterization of L34T fibrillization products, highlighting a marked polymorphism.

Starting as early as day 1 of aggregation, our observations revealed well-separated straight twisted fibers with highly periodic morphologies, and regularly spaced cross-over distances, leading to the identification of 2 groups of fibers depending on their axial periodicity (Figures 3E,H). Long cross-over distances of ∼ 105 nm are observed at all times over a 10-day period (Figures 3E–G), and are resembling to previously described periodic Aβ_1__–__42_ WT fibers (Schmidt et al., 2009; Young et al., 2017; Watanabe-Nakayama and Ono, 2018). Shorter cross-over distances are also observed, but evolved with time, increasing from ∼40 nm at day 1 to ∼60 nm at day 7 and ∼70 nm at day 10 of incubation (Figures 3H–J). At day 7 of incubation, twisted fibers are co-existing with non-twisted ones, though the later are a minority in our samples (Figures 3A,B), and curved fibers are also present at day 10 in a low proportion (Figure 3C), which is consistent with previously observed mature Aβ_1__–__42_ WT fibers (Lin et al., 2020).

### Spectroscopic Characterization of the Fibrillization Products at the Nanoscale

**FIGURE 4:**
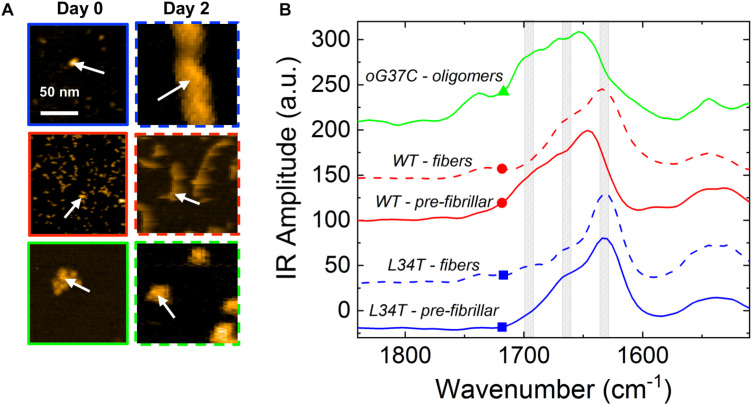
Representative NanoIR spectra coming from Aβ_1–42_ peptide variants in early and late stages of aggregation. **(A)** Representative AFM images of isolated structures of the different Aβ_1–42_ peptides variants for pre-fibrillar or fibrillar species obtained after 0 or 2 days of fibrillation, respectively. **(B)** Associated representative NanoIR spectra, acquired locally on the isolated structures. The WT peptides are represented in red, L34T in blue, and oG37C in green. Gray bands centered at 1630, 1660 and 1690 cm^–1^ indicate, respectively, parallel β-sheet, β-turn, and anti-parallel β-sheet. White arrows on **(A)** represent the position of the NanoIR tip when acquiring NanoIR spectra.

NanoIR measurements were performed on the different peptide variants to access their secondary structures at the nanoscale (Dazzi et al., 2012; Ruggeri et al., 2015; Henry et al., 2018), both in early stages of aggregation, i.e., on freshly thawed samples hereafter named pre-fibrillar samples, and later in the process after 2 days of incubation. This is of prime importance as it is now widely accepted that the toxicity of the amyloids and their secondary structures are linked (Vignaud et al., 2013; Bonhommeau et al., 2017). In particular, the high toxicity of some peptides could be due to the existence of oligomeric species organized in anti-parallel β-sheets (Seilheimer et al., 1997; Henry et al., 2015). Nevertheless, most studies are using characterization methods such as ATR-FTIR, Raman spectroscopy or Circular Dichroism, which have the drawback of averaging the spectral signatures of the different species present in the samples. As most of the time, the different forms of the peptides co-exist (monomers, oligomers, fibers, fibrils), it is difficult to get a precise information on the secondary structures of the different peptides. One of the major interests of NanoIR is that the IR spectra can be locally collected from isolated objects as shown on Figure 4, thus giving a reliable description of their secondary structures. NanoIR spectra shown in the Figure 4B were recorded from isolated representative species of each peptide both on pre-fibrillar samples, i.e., a heterogeneous mixture of monomers and small oligomers for WT and L34T *vs* a homogeneous population of oligomers for oG37C, and on 2-day samples, i.e., fibers for L34T and WT and still oligomers for oG37C. The oligomer oG37C is indeed stable in its oligomeric form and corresponds to a 14-mer (Bobo et al., 2017). The focus was given to the Amide I band between 1600 and 1700 cm^–1^ as it allowed discriminating β-sheets, parallel and antiparallel, as well as random coil secondary structures.

The nanoIR spectra obtained for *fibrillar* L34T and WT (Figure 4B, dashed lines, blue and red, respectively) show that both have a similar secondary structure with two main bands characteristic of a parallel β-sheet secondary structure around 1630 cm^–1^, which is the major component, and of a β-turn structure around 1660 cm^–1^. Regarding the oG37C oligomer, the IR spectrum is clearly different with the appearance of a shoulder with an important intensity around 1690 cm^–1^ and a clear decrease of the intensity of the 1630 cm^–1^ band. This new 1690 cm^–1^ band is characteristic of the anti-parallel β-sheet secondary structure. These results acquired on individual objects are consistent with previous ATR-FTIR (Vignaud et al., 2013) and Raman measurements (Bonhommeau et al., 2017). We also probed the secondary structure of *pre-fibrillar* WT and L34T species (Figure 4B, solid lines, red and blue, respectively) in order to assess the evolution of secondary structure over the course of the fibrillization process. Pre-fibrillar L34T exhibits a very similar IR signature compared to its fibrillar counterpart, with a dominant band characteristic of a parallel β-sheet secondary structure around 1630 cm^–1^, and a β-turn structure around 1660 cm^–1^. The early acquisition of a parallel β-sheet secondary structure for pre-fibrillar L34T is consistent with the fast fibrillization observed. However, the IR signature of pre-fibrillar WT slightly differs from fibrillar WT peptides, with a shift toward higher wavenumbers and a contribution in the 1690 cm^–1^ area, characteristic of the anti-parallel β-sheet secondary structure and close to the signature observed for oligomeric oG37C peptides. This indicates the presence of an anti-parallel β-sheet secondary structure in pre-fibrillar WT amyloid species, and suggests a reorganization over the course of the fibrillization process, as no anti-parallel β-sheet structure is observed in fibrillar WT species.

### Interaction of Amyloid Peptides and Fibers With Model Membranes

In order to assess the behavior of Aβ_1__–__42_ peptide variants on lipid membranes, real-time HS-AFM imaging of model membranes was performed before and after injection of freshly thawed Aβ_1__–__42_ L34T, WT, and oG37C peptides *in operando*. Thus, our objective is to assess a correlation between the peptide’s structure, their fibrillization behavior and their interaction with membranes, with potential deleterious effects. Control experiments have been performed in order to ensure that the scanning of model membranes by the AFM tip in our experimental conditions did not impact the integrity or the behavior of the supported lipid layers over long time periods largely higher than our timescale of observation (see Supplementary Video 1). When possible, areas at the membrane/mica interface were targeted in order to characterize the effect of peptides on the model membrane while assessing its interaction with the substrate. In addition, selected areas were first scanned for 10 min before injecting the peptide to ensure the stability of the membranes. The injection of the peptide is performed *in operando* in the experimental volume, and therefore the homogeneity in the fluid cell and the local concentration of peptides are not guaranteed at the moment of the scan, which explains the observed variations for the kinectics from one experiment to the other, and strongly motivated the repetition of experiments, in addition to occasional injections of peptides in larger quantities to confirm the effects.

**FIGURE 5:**
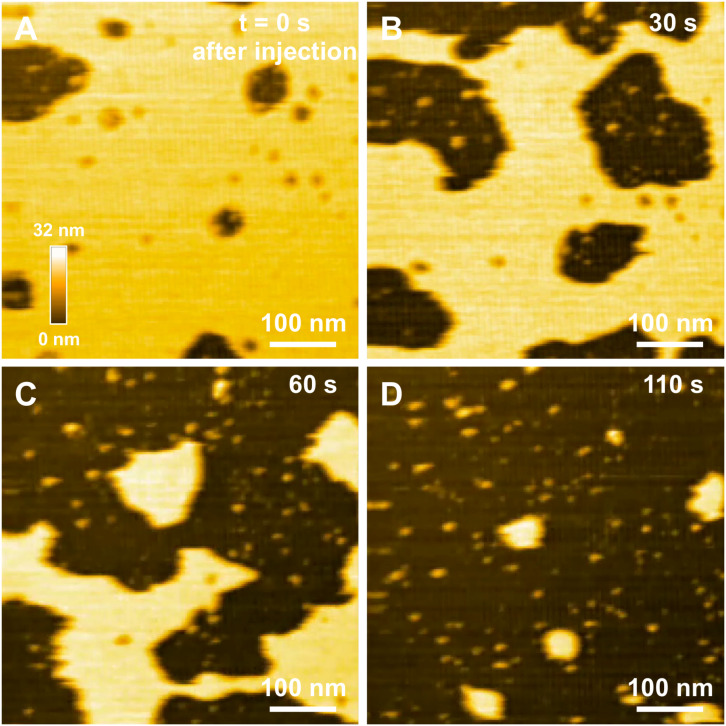
Interaction between oG37C peptides and a POPC/SM/Chol/GM1 membrane. Successive HS-AFM height images extracted from video 2 at the time of injection **(A)**, 30 s after injection **(B)**, 60 s after injection **(C)**, and 110 s after injection **(D)**.

**FIGURE 6:**
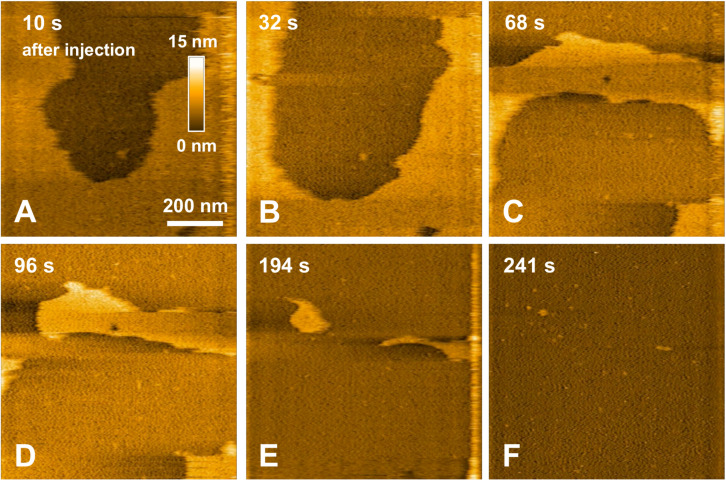
Interaction between pre-fibrillar WT peptides and a POPC/SM/Chol/GM1 membrane. Successive HS-AFM height images extracted from video 3 10 s after injection **(A)**, 32 s after injection **(B)**, 68 s after injection **(C)**, 96 s after injection **(D)**, 194 s after injection **(E)**, and 241 s after injection.

**FIGURE 7:**
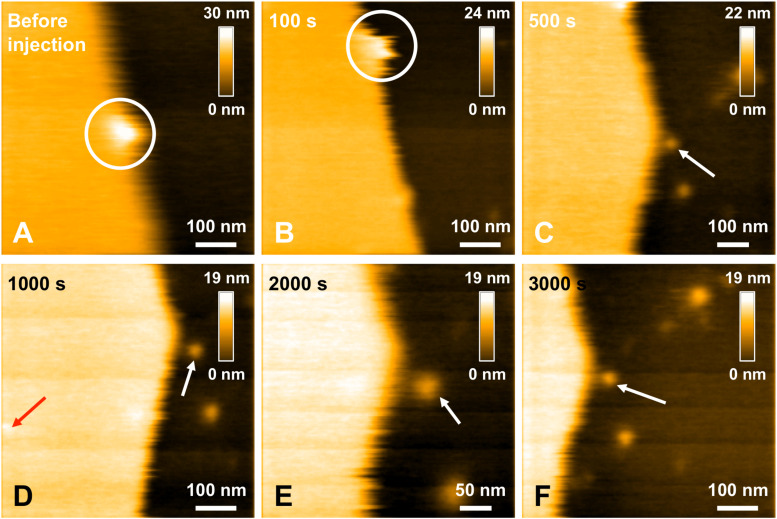
Interaction between L34T mutant peptides and a POPC/SM/Chol/GM1 membrane. Successive HS-AFM height images extracted from video 4 before injection **(A)**, 100 s after injection **(B)**, 500 s after injection **(C)**, 1000 s after injection **(D)**, 2000 s after injection **(E)**, and 3000 s after injection. The white circle indicates a same recognizable landmark in the scanned area to indicate a sight displacement of the scanned area toward the lower area over the course of the scan. Red and white arrows indicates L34T peptides in contact with the SLB or the mica substrate, respectively. One of the advantages of HS-AFM is the ability of moving the scanned area in real time, in the limits of the X and Y piezoelectric scanners, respectively ∼1 and 3.2 μm. This allows zooming in in real time to focus on details (as here in **E**).

In a previous study focusing on the toxic behavior of oligomeric amyloid peptides, the oG37C oligomer was used as a model peptide to unravel oligomer/membrane interactions (Ewald et al., 2019). Both cholesterol and GM1 are required in the membrane in order to observe oligomer-induced deleterious effects, with a fast and total dissolution of SM/PC/Chol/GM1 membranes (Ewald et al., 2019). As presented in Figure 5 (see Supplementary Video 1), upon addition of the oligomeric oG37C peptide in the experimental volume, the POPC/SM/Cholesterol/GM1 membrane coverage rapidly decreases and only small patches of membrane remain after 110 seconds. In agreement with previous work on membrane disruption by oG37C (Ewald et al., 2019) or by phospholipase D for instance (El Kirat et al., 2008), a marked enlargement of the pre-existing defects of the membrane was observed, and the membrane dissolution seemed to be driven at the edges of the SLB. This effect has been interpreted as membrane dissolution through a detergent effect induced by oG37C. After injection of Aβ_1__–__42_ WT peptides on a model SM/PC/Chol/GM1 membrane (Figure 6 and Supplementary Video 3), an extension of the holes present in the SLB is also evidenced, with progressive disappearance of the membrane until total dissolution after 240 s, indicating a strong interaction of the WT peptide with the membrane. As previously noticed for oG37C, no accumulation of peptide on the membrane or on the mica substrate was observed. The behavior of Aβ_1__–__42_ WT peptides on POPC/SM/Cholesterol/GM1 membranes is therefore similar to the one observed for toxic oligomers oG37C. This confirms that oG37C and its mechanisms of toxicity are representative of the WT peptide, even though its stable oligomeric state is originating from a purification based on size-selection (Vignaud et al., 2013). However, the fastest membrane dissolution observed for the WT peptide (Figure 6, 240 s) appears slower than for oG37C, where total dissolution was observed in under a minute (Ewald et al., 2019), even when taking into account the heterogeneity of kinetics between experiments. This is consistent with the WT peptide quickly starting to aggregate and form small oligomers when thawed and injected, with at least part of the oligomers formed adopting the structure and behavior of oG37C oligomers. In contrast to the WT and oG37C peptides, the injection of the L34T peptide did not induce a deleterious effect on the SM/PC/Chol/GM1 membrane. A slow accumulation of peptides on the mica substrate is observed, indicated on Figure 7 by white arrows (Figures 7C–F), while only few peptides (indicated by a red arrow, Figure 7D) are visible on the membrane after 1000 s. The density of peptides accumulating on the mica is, however, low (Figure 7F). In order to confirm our observations, a second injection was performed at *t* = 1750 s (noticeable on Supplementary Video 4), with no additional effect. L34T does not seem to interact with SM/PC/Chol/GM1 membranes, or only weakly.

**FIGURE 8:**
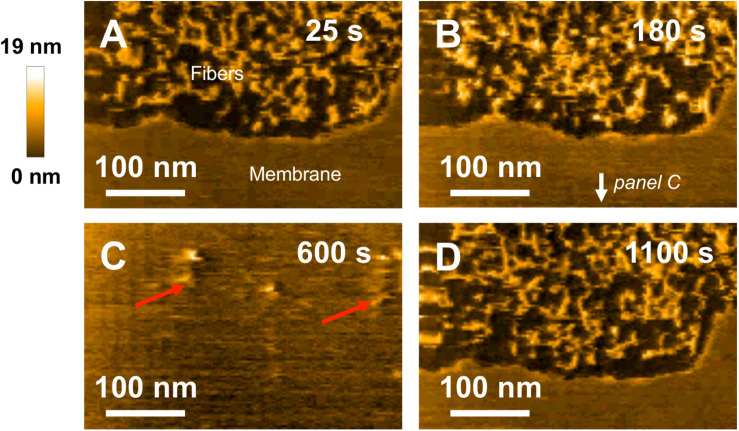
Interaction between fibrillar WT peptides and a POPC/SM/Chol/GM1 membrane. Successive HS-AFM height images extracted from video 5 25 s after injection **(A)**, 180 s after injection **(B)**, 600 s after injection, and 1100 s after injection. Fibers present on the membrance are pointed by red arrows on **(C)**.

Finally, the same experiment using fibrillar WT species was performed with HS-AFM to investigate their interaction with model SM/PC/Chol/GM1 membranes. 3Fibers and proto-fibrillar species quickly accumulate on the mica substrate after the injection (Figures 8A,B). Only a few fibrillar WT species are observed on the membrane (Figure 8C, indicated by red arrows), indicating weak interaction of fibrillar WT species with the membrane. Contrary to pre-fibrillar WT, which induced membrane dissolution (Figure 6), the integrity of the membrane is here preserved even 1100 s after injection of the fibrillar WT (Figure 8D and see Supplementary Video 5), thus confirming a weak interaction of fibrillar WT with the SM/PC/Chol/GM1 membranes.

Our HS-AFM experiments showed that in the same way as for the oG37C amyloid oligomer, the WT peptide interacted strongly with SM/PC/Chol/GM1 membranes during early stages of aggregation, with an amyloid-induced dissolution through a detergent effect. Small entities such as small oligomers are therefore responsible for this interaction in the pre-fibrillar stage of the aggregation process. Such detergent effect has already been observed in other studies (Williams and Serpell, 2011; Bode et al., 2019) in the case of Aβ oligomers. Aβ oligomers caused detergent-like disruption with lipid extraction and curvatures within PC/Chol/GM1 bilayers, leading to bilayer damage (Bode et al., 2019). A strong parallel can be drawn between Aβ oligomers and anti-microbial peptides, which show a similar detergent effect, which would depend on membrane insertion (Bechinger and Lohner, 2006). Nicastro et al. (2016) observed an insertion of Aβ peptides in liposomes with GM1-cholesterol domains, resulting in structural changes into the liposome internal layers. Our observations are in accordance with this model since the interaction of our oligomeric peptides (either oG37C or WT in the pre-fibrillar state) with SM/POPC/Chol/GM1 membrane leads to the rupture of the lipid membrane. Complementary ATR-FTIR measurements could allow quantifying A_β__1__–__42_ induced membrane solubilization. The challenging characterization of amyloid-lipid assemblies at the atomic level by mass and NMR spectrometries, combined to molecular dynamics simulations would allow establishing a detailed atomistic model of the mechanism at play. Recent studies have for instance allowed characterizing the formation of pores by Aβ_1__–__42_ tetramers and octamers (Ciudad et al., 2020) or the stability of Aβ_1__–__40_ trimers of parallel and antiparallel β-sheet structures (Ngo et al., 2020) in membrane mimicking environments. Interestingly, fibrillar WT no longer interacted with the membranes. Likewise, L34T only interacted weakly with the membrane, with no deleterious effect in the timescale of our experiments (∼3000 s of real-time observation). These different observations performed at the nanoscale are in favor of a negative correlation between fiber formation and interaction with membranes (Henry et al., 2015).

It could be noted that in our experiments, the disruption seems to start from the edges of the membranes: even if this effect concerns model membranes, such a disruption effect could also happen in living cells. Indeed, during the biological processes which would lead to interactions between peptides and cell membranes, some local forces could induce differences in the membrane morphology that could be assimilated to an edge in our experiments and this could explain *in vivo* membranes damages already observed elsewhere (Tarozzi et al., 2010; Yasumoto et al., 2019).

### Nanoscale IR Spectroscopy of Amyloid Peptides Interacting With Model Membranes

**FIGURE 9:**
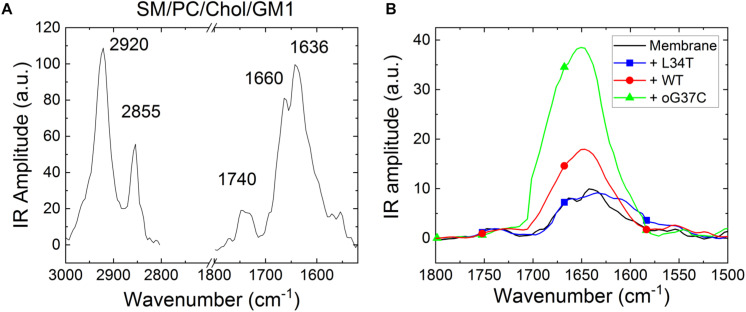
Representative NanoIR spectra coming from **(A)** SM/PC/Chol/GM1 model membrane and **(B)** pre-fibrillar Aβ_1–42_ peptides variants interacting with SM/PC/Chol/GM1 model membranes. The control membrane is in black, the WT peptide in red, L34T in blue, oG37C in green.

To get a better idea of the interactions between the peptides and the SM/PC/Chol/GM1 membranes, NanoIR measurements were carried out (Figures 9A,B). Such an approach is original as it could allow detecting the presence of the peptides through their spectroscopic signature, even if they are inserted within the membranes, which is a possibility regarding the HS-AFM experiments previously described. A previous study focused on the interaction of Aβ_1__–__42_ WT and oG37C peptides with SM/PC or SM/PC/Chol membranes (Henry et al., 2018), which allowed confirming the presence or the absence of the toxic peptides in the bilayers depending on the presence of cholesterol. In order to get enough sensitivity to chemically characterize thin model membranes with and without the peptides, as well as to increase the IR signal, we worked with lipid multilayers, typically 4 bilayers, i.e., model membranes of a thickness around 20 nm. An incubation time of ∼4 h after injection of the peptides was selected to let enough time for the peptides to interact with the membranes before performing our NanoIR observations. Because of the presence of multiple layers, not all of the membranes are degraded after 4 h, even in the case of the oG37C peptide. Topography of the model membranes was checked in liquid before injection, and after incubation before drying. As stated before, in the present study, the focus was made on SM/PC/Chol/GM1 membranes, as it is known that Cholesterol and GM1 are needed to lead to membrane degradation (Grimm et al., 2013; Ewald et al., 2019).

First, NanoIR experiments were performed on SM/PC/Chol/GM1 bilayers without peptides (Figure 9A) and results showed the presence of characteristic bands of organized lipids (Vigano et al., 2000). At high wavenumbers, two bands around 2920 and 2850 cm^–1^ are visible, which correspond to antisymmetric and symmetric CH_2_ vibration modes, respectively. In the Amide I area, two bands are also observed corresponding to the ester of PC around 1740 cm^–1^ and to the CO-amide groups of SM and GM1 around 1636 cm^–1^. As GM1 includes three amide groups but SM only one, the main contribution (at 1636 cm^–1^) comes from GM1 while the contribution of SM around 1660 cm^–1^ enlarges the IR band.

Once SM/PC/Chol/GM1 membranes were characterized, the same measurements were performed on these membranes after injection and incubation of freshly thawed peptides (L34T, WT, or oG37C), in order to evidence their possible interaction with the lipid layers (Figure 9B). Before drying the sample for NanoIR analysis, the samples were rinsed in order to remove non-interacting peptides still present in the solution. To highlight the effect of peptide injection, the focus was made on the amide I band for which the peptides could have an additional contribution (see Figure 4B). The spectra were then normalized regarding the CO stretching band around 1740 cm^–1^, as the main contribution of peptide addition should be observed in the 1600–1700 cm^–1^ range. Regarding the intensities and shapes of the amide I band, two clear behaviors were identified. For the L34T peptide, after injection, the spectra remain identical to the one of SM/PC/Chol/GM1 membranes and the representative bands coming from the peptides are not observed. It seems that the peptides are poorly interacting with the membrane, as rinsing the membrane before drying is sufficient to get rid of L34T peptides. For WT and oG37C peptides, the observed behavior is totally different as changes could be observed in the IR spectra, mostly regarding band intensity but also for band shape. Indeed, there is a strong increase in the intensity of the 1650 cm^–1^ band of about a factor 1.8 ± 0.4 for the WT peptide and 3.8 ± 0.5 for the oG37C oligomer, combined with the appearance of a higher wavelength component, especially for oG37C. The enlargement of the amide I band is due to the different secondary structures of WT and oG37C peptides that contribute to the IR signal, and is in line with the presence of the antiparallel β-sheet secondary structure of toxic peptides, centered at 1690 cm^–1^, as observed in isolated peptides in early stages of aggregation (Figure 4B). The two spectral changes observed indicate that the WT and oG37C peptides are accumulating on or within the membrane and that a strong interaction between WT and oG37C and SM/PC/Chol/GM1 membranes exists compared to L34T, as they are not removed by the rinsing step. These results confirmed the hypothesis previously made based on our HS-AFM imaging: WT and oG37C peptides that are leading to membrane disruption have a strong interaction with the membranes. They are surely present within or attached at the surface of the membranes contrary to the L34T peptides, which are not evidenced in/on the membranes after injection and drying of the samples. The results clearly establish a correlation between toxicity of the peptides, their interactions with SM/PC/Chol/GM1 membranes and membrane disruption.

## Discussion

In this study, a series of mutant Aβ_1__–__42_ peptides were studied and their link at the nanoscale between their fibrillization kinetics, morphologies and structure, their interaction with membranes and membrane toxicity were investigated. To that end, it was taken advantage of the high spatial and temporal resolution of HS-AFM, as well as the exciting nanoscale chemical possibilities of NanoIR.

Marked differences in fibrillization kinetics and morphology of fibrillization products for the 3 considered Aβ_1__–__42_ peptides were evidenced. The L34T peptides grew more rapidly than its WT analog, with the acquisition of twisted morphologies with highly periodic structures as early as day 1. This abundant twisted morphology has been shown to be promoted by the presence of NaCl in the growth medium, whereas KCl would promote an alternation between twisted and straight/smooth morphologies (Watanabe-Nakayama and Ono, 2018). It is therefore related to assembly conditions. The observed co-existence of long and short periodicities was previously observed for Aβ_1__–__40_ fibers (Goldsbury et al., 2005), with two predominant mature fiber types, coiled fibrils with short cross-over spacing of ∼ 25 nm and twisted ribbons with longer periodicities ranging from 80 to 130 nm, which was interpreted as multiple assembly pathways. Recent cryo-EM data on Aβ polymorphism indicated three dominant types of fibrils, with varied axial periodicities involving an increasing number of protofilaments in the fibril (Kollmer et al., 2019). With one protofilament, the first fibril morphology presented short cross-over distances of ∼ 41.5 nm close to the one observed at day 1 of fibrillization. A second and third morphology presented 2 and 3 protofilaments, with increasing cross-over distances of ∼129 and 143 nm, respectively. However, in contrast to our results, this increase in cross-over distance was accompanied with an increase in width, which we do not observe, thus ruling out an increase in the number of protofilaments as the cause of the observed twist polymorphism in our L34T samples. Indeed, most of our observed L34T fibers seemed to be made of single protofilaments, even though we occasionally observed mature fibrils with 2 twisted protofilaments (day 10, Figure 3D), in agreement with previous results (Young et al., 2017; Cendrowska et al., 2020). The modification of the helical step of formed fibers could indicate a rearrangement of protofibrils during the formation of mature fibrils, a reorganization of β sheets as times goes, as shown by ATR-FTIR (Sarroukh et al., 2013). Interestingly, amyloid aggregates isolated from brain or heart exhibit morphological homogeneity, notably in term of helical periodicity (Cendrowska et al., 2020). Though a majority of the observed L34T fibers showed a left-handed twist, in agreement with most *in vitro* grown fibers (Kollmer et al., 2019), occasional right-handed ones as pictured in Figure 2B were observed. An increasing structural complexity during fiber thickening could explain the inversion of handedness as for short amphiphilic peptides (Wang et al., 2017). Yet both fibers on our image (Figure 2B) are of similar width. The co-existence of both morphologies could, however, be explained by the possibility for protofilaments to adopt varied twist angles from −11 on the left side to +8 degrees on the right side in response to external conditions (Periole et al., 2018). The possibility for several left-handed fibrils to assemble into a right-handed one could also explain this observation (Wang et al., 2017). Compared to L34T, the WT peptide formed well-structured fibers on a longer timescale. The WT fibrils observed at day 7 of incubation, shorter and thicker than the L34T fibers, resemble the striated ribbon fibrils described for Aβ_1__–__40_, involving a variable number of protofilaments (Tycko, 2015). Finally, the oG37C peptide did not fibrillate, thus confirming its stability as an oligomer.

NanoInfrared measurements on isolated peptide structures confirmed the high content of all probed amyloid peptides in β-sheet secondary structures. Going into details, the presence of anti-parallel β-sheet secondary structures in oG37C peptides was evidenced with a strong contribution at 1690 cm^–1^ in the nanoIR spectra, in agreement with previous work leading to averaged signals using circular dichroism and ATR-FTIR (Vignaud et al., 2013) and with existing literature (Cerf et al., 2009). In addition, NanoIR allowed to evidence a structural difference in the WT peptide depending on its aggregation stage. Fibrillar WT, like pre-fibrillar and fibrillar L34T, showed classic amyloid IR signatures, with a dominant parallel β-sheet secondary structure. In contrast, WT species in the early stage of aggregation exhibited a clear contribution of anti-parallel β-sheet secondary structure. This implies that (i) pre-fibrillar WT includes anti-parallel aggregates, often associated with toxic species, and (ii) the secondary structure of the WT peptides evolves in time, with a re-organization to obtain parallel structures in the final fibrillization product. These observations are in full agreement with previous characterization of Aβ_1__–__42_ by ATR-FTIR (Cerf et al., 2009), as well as with the structural characterization by NanoIR of α-synuclein oligomers and fibers at different aggregation stages (Zhou and Kurouski, 2020), with spherical aggregates always exhibiting a higher anti-parallel β-sheet content than the fibrillar ones. Furthermore, a recent study combining SAXS and molecular dynamics showed that the first stages of aggregation, with small length- and time-scales, would be dominated by antiparallel structures, even if later stages in the aggregation exhibited the parallel β-sheet as the most common structure (Zanjani et al., 2020).

The link between antiparallel β structure and oligomer toxicity is more and more documented in literature. For instance, it was shown by ATR-FTIR that whey proline-rich peptides suppressed both oligomerization and the formation of antiparallel β-sheet (Bharadwaj et al., 2013), leading to a rescue of yeast and neuronal cells. Such modulation of the folding pathway of Aβ_1__–__42_ has also been shown for Fucosterol, which protected neuronal cells against Aβ_1__–__42_-induced toxicity (Gan et al., 2019). Using ThT fluorescence, AFM, and molecular dynamics, Castro-Silva and co-workers demonstrated that fucosterol inhibits Aβ_1__–__42_ aggregation by binding to structural regions of the monomers involved in the formation of antiparallel β strand of the Aβ_1__–__42_ oligomer (Castro-Silva et al., 2020). In addition to strengthening the link between antiparallel β structure and oligomer toxicity, the present study allows linking both parameters to the interaction of the oligomers with membrane. HS-AFM experiments of membranes interacting with variant amyloid peptides, as well as their chemical characterization by NanoIR, allowed assessing of (i) the strength of the amyloid/membrane interaction and (ii) the membrane toxicity of each of the L34T, WT and oG37C peptides through the assessment of membrane damage. Though fiber formation is enhanced for L34T, it interacted only weakly with the membrane, as it did not contribute to the IR spectra obtained after incubation. Moreover, it did not induce any membrane damage, and barely accumulated on the membrane. In Aβ_1__–__40_, the Leucine residue in position 34 is involved in molecular contact with a phenylalanine residue in position 19. This contact has been shown to influence oligomer stability, fibril elongation and local structures, and substitutions of L34 have led to a decrease in toxicity, while substituting the F19 residue completely abolished the toxicity (Korn et al., 2018). A double mutant of Aβ_1__–__42_ with F19S and L34P substitutions has even presented protective effects against Aβ_1__–__42_ induced phospholipid membrane destabilization and permeation (Oren et al., 2020). This is consistent with the results obtained in the present study for the L34T peptide, where substituting a Leucine by a Threonine residue in position 34 led to a lower membrane interaction and no membrane damage. In contrast, two distinct sets of characteristics for the WT peptides were highlighted in this study, depending on the aggregation stage. In early stages of fibrillization, NanoIR spectroscopy showed strong interaction between pre-fibrillar WT species and model SM/PC/Chol/GM1 membranes. Moreover, the injection of pre-fibrillar WT species on the membrane led to a disruption of the membrane through a detergent effect, thus presenting a deleterious behavior similar to the one described for the oG37c oligomers (Ewald et al., 2019) and consistent with recent literature on Aβ_1__–__42_ interacting with PC/GM1/Chol membranes (Bode et al., 2019). This confirmed a strong interaction of small WT entities with the membranes in the early stages of aggregation. However, in later stages of aggregation, fibrillar WT species did not interact with the membrane, and had no deleterious effect on the membrane, in agreement with recent AFM and electron microscopy studies (Bode et al., 2019) and consistent with weak interactions with the membrane. This study deepens the fundamental understanding of how amyloid species interact with membranes, yet we are still far from addressing how this damage mechanism of Aβ-induced detergent-like disruption of the membrane ties into the development of Alzheimer’s disease. From our model lipid membrane system to a complex cellular environment, many additional processes will need to be taken into account, from the role of membrane proteins in the Aβ/membrane interaction to existing membrane repair processes (Jimenez et al., 2014).

**FIGURE 10:**
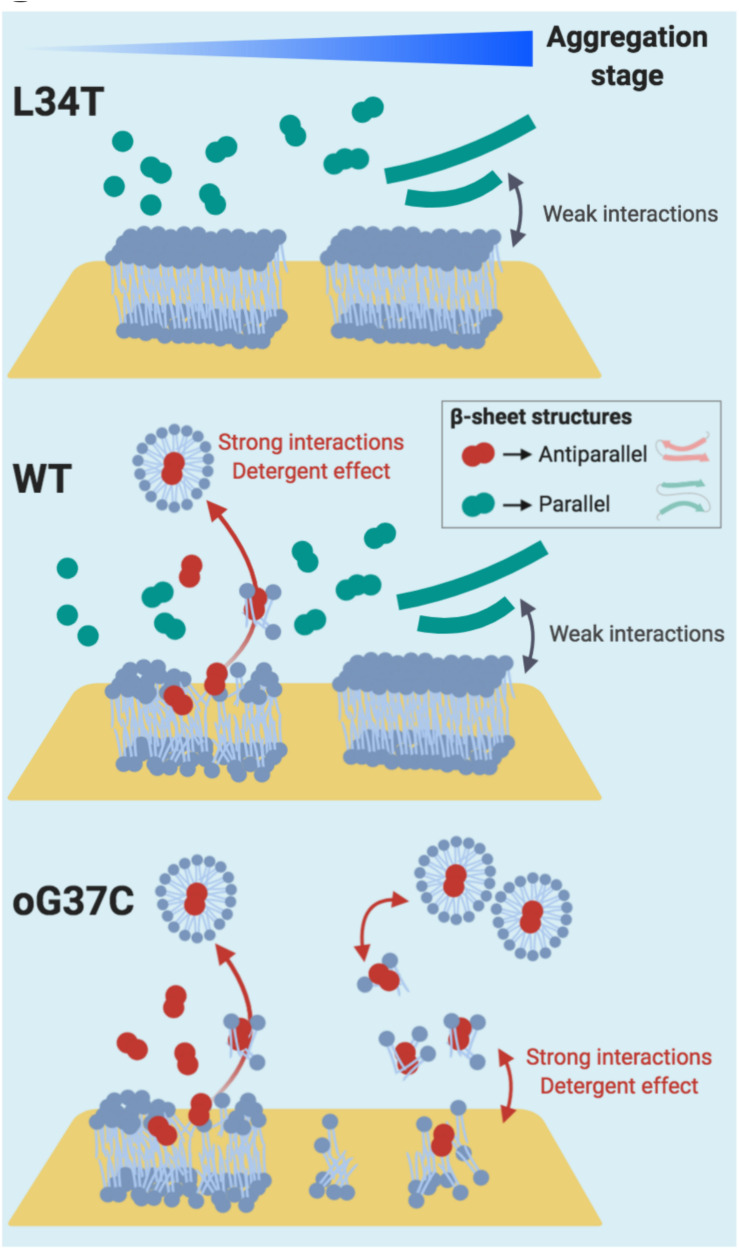
Proposed Interaction schemes between Aβ1-42 L34T, WT, and oG37C peptide and POPC/SM/Chol/GM1 model membranes in early and late stages of amyloid aggregation. Anti-parallel β sheet structures are figured in red, while parallel β sheet structures are figured in green. Based on our observations in HS-AFM and NanoIR spectroscopy, strong interactions between the amyloid peptides and the membrane are only observed in the early stages of fibrillation, i.e., with small oligomers, and coincide with the presence of anti-parallel β sheet structures. These anti-parallel β sheet structures, similar to the oG37C toxic oligomer, would induce the dissolution of the membrane through a detergent effect.

In summary (Figure 10), considering our HS-AFM and NanoIR data, it can be concluded that membrane disruption is associated to amyloid species that (i) present strong interactions with the membrane, as well as (ii) an antiparallel β-sheet secondary structure, and (iii) are only transient small oligomeric entities in the early stages of aggregation. The strong interaction between oligomeric species and the membrane could therefore be a valuable target to consider in order to inhibit Aβ_1__–__42_ toxicity. A recent study focused on both reducing the binding affinity of oligomers to membranes and enhancing aggregation (Limbocker et al., 2019), therefore promoting the conversion from toxic oligomer to less toxic fibrillar species, with promising results. The present study provides a comprehensive set of data linking aggregation, structure, interactions with membranes and deleterious effects on membranes, thus deepening our understanding of the amyloid/membrane interaction.

## Data Availability Statement

The raw data supporting the conclusions of this article will be made available by the authors, upon request.

## Author Contributions

CC designed and prepared the different peptide mutants. CF, MA, EL, and SH prepared the model membranes. EL and ME carried out the HS-AFM experiments. CF and MM performed the NanoIR experiments. EL, MA, and SM performed the AFM experiments to check the sample integrity. CF, EL, ME, and MM analyzed and treated the data. CF and MM prepared the figures and the manuscript. CF, MM, EL, ME, SH, MA, SM, CC, and SL corrected the manuscript. SL and MM designed the study. All authors contributed to the article and approved the submitted version.

## Conflict of Interest

The authors declare that the research was conducted in the absence of any commercial or financial relationships that could be construed as a potential conflict of interest.

## References

[B1] Adamcik J. Mezzenga R. (2018). Amyloid polymorphism in the protein folding and aggregation energy landscape. *Angew. Chem.* 57 8370–8382. 10.1002/anie.201713416 29446868

[B2] Ando T. Kodera N. Takai E. Maruyama D. Saito K. Toda A. (2001). A high-speed atomic force microscope for studying biological macromolecules. *Proc. Natl. Acad. Sci. U.S.A.* 98 12468–12472. 10.1073/pnas.211400898 11592975 PMC60077

[B3] Azouz M. Cullin C. Lecomte S. Lafleur M. (2019). Membrane domain modulation of Aβ1–42 oligomer interactions with supported lipid bilayers: an atomic force microscopy investigation. *Nanoscale* 11 20857–20867. 10.1039/c9nr06361g 31657431

[B4] Bechinger B. Lohner K. (2006). Detergent-like actions of linear amphipathic cationic antimicrobial peptides. *Biochim. Biophys. Acta* 1758 1529–1539. 10.1016/j.bbamem.2006.07.001 16928357

[B5] Berthelot K. Cullin C. Lecomte S. (2013). What does make an amyloid toxic: morphology, structure or interaction with membrane? *Biochimie* 95 12–19. 10.1016/j.biochi.2012.07.011 22824150

[B6] Bharadwaj P. Head R. Martins R. Raussens V. Sarroukh R. Jegasothy H. (2013). Modulation of amyloid-beta 1-42 structure and toxicity by proline-rich whey peptides. *Food Funct.* 4 92–103. 10.1039/c2fo30111c 23014463

[B7] Bobo C. Chaignepain S. Henry S. Vignaud H. Ameadan A. Marchal C. (2017). Synthetic toxic Abeta1-42 oligomers can assemble in different morphologies. *Biochim. Biophys. Acta* 1861(5 Pt A), 1168–1176. 10.1016/j.bbagen.2017.03.001 28267577

[B8] Bode D. C. Freeley M. Nield J. Palma M. Viles J. H. (2019). Amyloid-beta oligomers have a profound detergent-like effect on lipid membrane bilayers, imaged by atomic force and electron microscopy. *J. Biol. Chem.* 294 7566–7572. 10.1074/jbc.ac118.007195 30948512 PMC6514634

[B9] Bonhommeau S. Lecomte S. (2018). Tip-enhanced Raman spectroscopy: a tool for nanoscale chemical and structural characterization of biomolecules. *Chemphyschem* 19 8–18. 10.1002/cphc.201701067 29106771

[B10] Bonhommeau S. Talaga D. Hunel J. Cullin C. Lecomte S. (2017). Tip-enhanced Raman spectroscopy to distinguish toxic oligomers from Abeta1-42 fibrils at the nanometer scale. *Angew. Chem.* 56 1771–1774. 10.1002/anie.201610399 28071842

[B11] Castro-Silva E. S. Bello M. Rosales-Hernandez M. C. Correa-Basurto J. Hernandez-Rodriguez M. Villalobos-Acosta D. (2020). Fucosterol from *Sargassum horridum* as an amyloid-beta (Abeta1-42) aggregation inhibitor: *in vitro* and *in silico* studies. *J. Biomol. Struct. Dyn.* 10.1080/07391102.2020.1729863 32159448

[B12] Cendrowska U. Silva P. J. Ait-Bouziad N. Muller M. Guven Z. P. Vieweg S. (2020). Unraveling the complexity of amyloid polymorphism using gold nanoparticles and cryo-EM. *Proc. Natl. Acad. Sci. U.S.A.* 117 6866–6874. 10.1073/pnas.1916176117 32161130 PMC7104366

[B13] Cerf E. Sarroukh R. Tamamizu-Kato S. Breydo L. Derclaye S. Dufrene Y. F. (2009). Antiparallel beta-sheet: a signature structure of the oligomeric amyloid beta-peptide. *Biochem. J.* 421 415–423. 19435461 10.1042/BJ20090379

[B14] Ciudad S. Puig E. Botzanowski T. Meigooni M. Arango A. S. Do J. (2020). Aβ(1-42) tetramer and octamer structures reveal edge conductivity pores as a mechanism for membrane damage. *Nat. Commun.* 11:3014. 10.1038/s41467-020-16566-1 PMC7296003 32541820

[B15] Dai Y. Zhang M. Shi X. Wang K. Gao G. Shen L. (2020). Kinetic study of Abeta(1-42) amyloidosis in the presence of ganglioside-containing vesicles. *Colloids Surf. B Biointerfaces* 185:110615. 10.1016/j.colsurfb.2019.110615 31707229

[B16] D’Angelo F. Vignaud H. Di Martino J. Salin B. Devin A. Cullin C. (2013). A yeast model for amyloid-beta aggregation exemplifies the role of membrane trafficking and PICALM in cytotoxicity. *Dis. Models Mech.* 6 206–216. 10.1242/dmm.010108 22888099 PMC3529352

[B17] Dazzi A. Glotin F. Carminati R. (2010). Theory of infrared nanospectroscopy by photothermal induced resonance. *J. Appl. Phys.* 107:124519 10.1063/1.3429214

[B18] Dazzi A. Prater C. B. Hu Q. Chase D. B. Rabolt J. F. Marcott C. (2012). AFM-IR: combining atomic force microscopy and infrared spectroscopy for nanoscale chemical characterization. *Appl. Spectrosc.* 66 1365–1384. 10.1366/12-06804 23231899

[B19] Doig A. J. Del Castillo-Frias M. P. Berthoumieu O. Tarus B. Nasica-Labouze J. Sterpone F. (2017). Why is research on amyloid-beta failing to give new drugs for Alzheimer’s disease? *ACS Chem. Neurosci.* 8 1435–1437. 10.1021/acschemneuro.7b00188 28586203

[B20] Drolle E. Hane F. Lee B. Leonenko Z. (2014). Atomic force microscopy to study molecular mechanisms of amyloid fibril formation and toxicity in Alzheimer’s disease. *Drug Metab. Rev.* 46 207–223. 10.3109/03602532.2014.882354 24495298

[B21] Drolle E. Negoda A. Hammond K. Pavlov E. Leonenko Z. (2017). Changes in lipid membranes may trigger amyloid toxicity in Alzheimer’s disease. *PLoS One* 12:e0182194. 10.1371/journal.pone.0182194 28767712 PMC5540602

[B22] El Kirat K. Dupres V. Dufrene Y. F. (2008). Blistering of supported lipid membranes induced by Phospholipase D, as observed by real-time atomic force microscopy. *Biochim. Biophys. Acta* 1778 276–282. 10.1016/j.bbamem.2007.09.029 17963688

[B23] Ewald M. Henry S. Lambert E. Feuillie C. Bobo C. Cullin C. (2019). High speed atomic force microscopy to investigate the interactions between toxic Abeta1-42 peptides and model membranes in real time: impact of the membrane composition. *Nanoscale* 11 7229–7238. 10.1039/c8nr08714h 30924478

[B24] Fitzpatrick A. W. Debelouchina G. T. Bayro M. J. Clare D. K. Caporini M. A. Bajaj V. S. (2013). Atomic structure and hierarchical assembly of a cross-beta amyloid fibril. *Proc. Natl. Acad. Sci. U.S.A.* 110 5468–5473. 23513222 10.1073/pnas.1219476110 PMC3619355

[B25] Fruhmann G. Marchal C. Vignaud H. Verduyckt M. Talarek N. De Virgilio C. (2018). The impact of ESCRT on Abeta1-42 induced membrane lesions in a yeast model for Alzheimer’s disease. *Front. Mol. Neurosci.* 11:406. 10.3389/fnmol.2018.00406 30455629 PMC6230623

[B26] Gan S. Y. Wong L. Z. Wong J. W. Tan E. L. (2019). Fucosterol exerts protection against amyloid beta-induced neurotoxicity, reduces intracellular levels of amyloid beta and enhances the mRNA expression of neuroglobin in amyloid beta-induced SH-SY5Y cells. *Int. J. Biol. Macromol.* 121 207–213. 10.1016/j.ijbiomac.2018.10.021 30300695

[B27] Goldsbury C. Frey P. Olivieri V. Aebi U. Muller S. A. (2005). Multiple assembly pathways underlie amyloid-beta fibril polymorphisms. *J. Mol. Biol.* 352 282–298. 10.1016/j.jmb.2005.07.029 16095615

[B28] Grimm M. O. W. Zimmer V. C. Lehmann J. Grimm H. S. Hartmann T. (2013). The impact of cholesterol, DHA, and sphingolipids on Alzheimer’s disease. *Biomed Res. Int.* 2013:814390. 10.1155/2013/814390 PMC3929518 24575399

[B29] Hane F. T. Lee B. Y. Petoyan A. Rauk A. Leonenko Z. (2014). Testing synthetic amyloid-beta aggregation inhibitor using single molecule atomic force spectroscopy. *Biosens. Bioelectron.* 54 492–498. 10.1016/j.bios.2013.10.060 24321883

[B30] Henry S. Bercu N. B. Bobo C. Cullin C. Molinari M. Lecomte S. (2018). Interaction of Abeta1-42 peptide or their variant with model membrane of different composition probed by infrared nanospectroscopy. *Nanoscale* 10 936–940. 10.1039/c7nr07489a 29292465

[B31] Henry S. Vignaud H. Bobo C. Decossas M. Lambert O. Harte E. (2015). Interaction of Abeta(1-42) amyloids with lipids promotes “off-pathway” oligomerization and membrane damage. *Biomacromolecules* 16 944–950. 10.1021/bm501837w 25689632

[B32] Islam Z. Ali M. H. Popelka A. Mall R. Ullah E. Ponraj J. (2020). Probing the fibrillation of lysozyme by nanoscale-infrared spectroscopy. *J. Biomol. Struct. Dyn.* 10.1080/07391102.2020.1734091 [Epub ahead of print]. 32131712

[B33] Jimenez A. J. Maiuri P. Lafaurie-Janvore J. Divoux S. Piel M. Perez F. (2014). ESCRT machinery is required for plasma membrane repair. *Science* 343:1247136. 10.1126/science.1247136 24482116

[B34] Kepp K. P. (2017). Ten challenges of the amyloid hypothesis of Alzheimer’s disease. *J. Alzheimers Dis.* 55 447–457. 10.3233/jad-160550 27662304

[B35] Kollmer M. Close W. Funk L. Rasmussen J. Bsoul A. Schierhorn A. (2019). Cryo-EM structure and polymorphism of Abeta amyloid fibrils purified from Alzheimer’s brain tissue. *Nat. Commun.* 10:4760. 10.1038/s41467-019-12683-8 PMC6820800 31664019

[B36] Korn A. McLennan S. Adler J. Krueger M. Surendran D. Maiti S. (2018). Amyloid beta (1-40) toxicity depends on the molecular contact between phenylalanine 19 and leucine 34. *ACS Chem. Neurosci.* 9 790–799. 10.1021/acschemneuro.7b00360 29232098

[B37] Limbocker R. Chia S. Ruggeri F. S. Perni M. Cascella R. Heller G. T. (2019). Trodusquemine enhances Abeta42 aggregation but suppresses its toxicity by displacing oligomers from cell membranes. *Nat. Commun.* 10:225. 10.1038/s41467-018-07699-5 PMC6333784 30644384

[B38] Lin D. Lei J. Li S. Zhou X. Wei G. Yang X. (2020). Investigation of the dissociation mechanism of single-walled carbon nanotube on mature amyloid-beta fibrils at single nanotube level. *J. Phys. Chem. B* 124 3459–3468. 10.1021/acs.jpcb.0c00916 32283926

[B39] Mastrangelo I. A. Ahmed M. Sato T. Liu W. Wang C. Hough P. (2006). High-resolution atomic force microscopy of soluble Abeta42 oligomers. *J. Mol. Biol.* 358 106–119. 16499926 10.1016/j.jmb.2006.01.042

[B40] Matsuzaki K. (2014). How do membranes initiate Alzheimer’s disease? Formation of toxic amyloid fibrils by the amyloid beta-protein on ganglioside clusters. *Acc. Chem. Res.* 47 2397–2404. 10.1021/ar500127z 25029558

[B41] Ngo S. T. Nguyen P. H. Derreumaux P. (2020). Stability of Aβ11–40 trimers with parallel and antiparallel β-sheet organizations in a membrane-mimicking environment by replica exchange molecular dynamics simulation. *J. Phys. Chem. B* 124 617–626. 10.1021/acs.jpcb.9b10982 31931566

[B42] Nicastro M. C. Spigolon D. Librizzi F. Moran O. Ortore M. G. Bulone D. (2016). Amyloid beta-peptide insertion in liposomes containing GM1-cholesterol domains. *Biophys. Chem.* 208 9–16. 10.1016/j.bpc.2015.07.010 26259785

[B43] Nirmalraj P. N. List J. Battacharya S. Howe G. Xu L. Thompson D. (2020). Complete aggregation pathway of amyloid beta (1-40) and (1-42) resolved on an atomically clean interface. *Sci. Adv.* 6:eaaz6014. 10.1126/sciadv.aaz6014 32285004 PMC7141833

[B44] Oren O. Ben Zichri S. Taube R. Jelinek R. Papo N. (2020). Abeta42 double mutant inhibits Abeta42-induced plasma and mitochondrial membrane disruption in artificial membranes, isolated organs, and intact cells. *ACS Chem. Neurosci.* 11 1027–1037. 10.1021/acschemneuro.9b00638 32155047

[B45] Panza F. Lozupone M. Logroscino G. Imbimbo B. P. (2019). A critical appraisal of amyloid-beta-targeting therapies for Alzheimer disease. *Nat. Rev. Neurol.* 15 73–88. 10.1038/s41582-018-0116-6 30610216

[B46] Periole X. Huber T. Bonito-Oliva A. Aberg K. C. van der Wel P. C. A. Sakmar T. P. (2018). Energetics underlying twist polymorphisms in amyloid fibrils. *J. Phys. Chem. B* 122 1081–1091. 10.1021/acs.jpcb.7b10233 29254334 PMC5857390

[B47] Ruggeri F. S. Longo G. Faggiano S. Lipiec E. Pastore A. Dietler G. (2015). Infrared nanospectroscopy characterization of oligomeric and fibrillar aggregates during amyloid formation. *Nat. Commun.* 6:7831. 10.1038/ncomms8831 PMC4525161 26215704

[B48] Sarroukh R. Goormaghtigh E. Ruysschaert J. M. Raussens V. (2013). ATR-FTIR: a “rejuvenated” tool to investigate amyloid proteins. *Biochim. Biophys. Acta* 1828 2328–2338. 10.1016/j.bbamem.2013.04.012 23746423

[B49] Sasahara K. Morigaki K. Shinya K. (2013). Effects of membrane interaction and aggregation of amyloid beta-peptide on lipid mobility and membrane domain structure. *Phys. Chem. Chem. Phys.* 15 8929–8939. 10.1039/c3cp44517h 23515399

[B50] Schmidt M. Sachse C. Richter W. Xu C. Fandrich M. Grigorieff N. (2009). Comparison of Alzheimer Abeta(1-40) and Abeta(1-42) amyloid fibrils reveals similar protofilament structures. *Proc. Natl. Acad. Sci. U.S.A.* 106 19813–19818. 10.1073/pnas.0905007106 19843697 PMC2764733

[B51] Scomparin C. Lecuyer S. Ferreira M. Charitat T. Tinland B. (2009). Diffusion in supported lipid bilayers: influence of substrate and preparation technique on the internal dynamics. *Eur. Phys. J. E Soft Matter* 28 211–220. 10.1140/epje/i2008-10407-3 19101741

[B52] Seilheimer B. Bohrmann B. Bondolfi L. Muller F. Stuber D. Dobeli H. (1997). The toxicity of the Alzheimer’s beta-amyloid peptide correlates with a distinct fiber morphology. *J. Struct. Biol.* 119 59–71. 10.1006/jsbi.1997.3859 9216088

[B53] Selkoe D. J. (2001). Alzheimer’s disease: genes, proteins, and therapy. *Physiol. Rev.* 81 741–766. 11274343 10.1152/physrev.2001.81.2.741

[B54] Tarozzi A. Morroni F. Merlicco A. Bolondi C. Teti G. Falconi M. (2010). Neuroprotective effects of cyanidin 3-O-glucopyranoside on amyloid beta (25-35) oligomer-induced toxicity. *Neurosci. Lett.* 473 72–76. 10.1016/j.neulet.2010.02.006 20152881

[B55] Tycko R. (2015). Amyloid polymorphism: structural basis and neurobiological relevance. *Neuron* 86 632–645. 10.1016/j.neuron.2015.03.017 25950632 PMC4425266

[B56] Vigano C. Manciu L. Buyse F. Goormaghtigh E. Ruysschaert J. M. (2000). Attenuated total reflection IR spectroscopy as a tool to investigate the structure, orientation and tertiary structure changes in peptides and membrane proteins. *Pept. Sci.* 55 373–380. 10.1002/1097-0282(2000)55:5<373::aid-bip1011>3.0.co;2-u 11241212

[B57] Vignaud H. Bobo C. Lascu I. Sorgjerd K. M. Zako T. Maeda M. (2013). A structure-toxicity study of Ass42 reveals a new anti-parallel aggregation pathway. *PLoS One* 8:e80262. 10.1371/journal.pone.0080262 24244667 PMC3823702

[B58] Wang M. Zhou P. Wang J. Zhao Y. Ma H. Lu J. R. (2017). Left or right: how does amino acid chirality affect the handedness of nanostructures self-assembled from short amphiphilic peptides? *J. Am. Chem. Soc.* 139 4185–4194. 10.1021/jacs.7b00847 28240550

[B59] Watanabe-Nakayama T. Ono K. (2018). High-speed atomic force microscopy of individual amyloidogenic protein assemblies. *Methods Mol. Biol.* 1814 201–212. 10.1007/978-1-4939-8591-3_12 29956234

[B60] Watanabe-Nakayama T. Ono K. Itami M. Takahashi R. Teplow D. B. Yamada M. (2016). High-speed atomic force microscopy reveals structural dynamics of amyloid beta1-42 aggregates. *Proc. Natl. Acad. Sci. U.S.A.* 113 5835–5840. 27162352 10.1073/pnas.1524807113 PMC4889376

[B61] Williams T. L. Serpell L. C. (2011). Membrane and surface interactions of Alzheimer’s Abeta peptide–insights into the mechanism of cytotoxicity. *FEBS J.* 278 3905–3917. 10.1111/j.1742-4658.2011.08228.x 21722314

[B62] Yasumoto T. Takamura Y. Tsuji M. Watanabe-Nakayama T. Imamura K. Inoue H. (2019). High molecular weight amyloid beta1-42 oligomers induce neurotoxicity via plasma membrane damage. *FASEB J.* 33 9220–9234. 10.1096/fj.201900604r 31084283

[B63] Young L. J. Kaminski Schierle G. S. Kaminski C. F. (2017). Imaging Abeta(1-42) fibril elongation reveals strongly polarised growth and growth incompetent states. *Phys. Chem. Chem. Phys.* 19 27987–27996. 10.1039/c7cp03412a 29026905 PMC7612976

[B64] Zanjani A. A. H. Reynolds N. P. Zhang A. Schilling T. Mezzenga R. Berryman J. T. (2020). Amyloid evolution: antiparallel replaced by parallel. *Biophys. J.* 118 2526–2536. 10.1016/j.bpj.2020.03.023 32311316 PMC7231890

[B65] Zhou L. Kurouski D. (2020). Structural characterization of individual alpha-synuclein oligomers formed at different stages of protein aggregation by atomic force microscopy-infrared spectroscopy. *Anal. Chem.* 92 6806–6810. 10.1021/acs.analchem.0c00593 32347706

